# The Art Amateur

**Published:** 1895-01

**Authors:** 


### The Art Amateur, Montague Marks, Publisher, 23 Union Square, New York. Price, 35 cents.

One can hardly imagine a more suitable Christmas gift for an artist friend than the December number of *The Art Amateur*. As it lies now before us it seems indeed a treasury of art, both for the art student—to whom it is a necessity—and the general reader. On opening it we find two charming color plates—absolute *fac similes* of costly paintings: "A Summer Evening," by E. Sanchez-Perrier, and "Purple and Gold" (Pansies), by Maud Stumm. Then there is a very large life-study in charcoal, printed on gray-blue paper, and eight pages of working designs for china and glass painting, embroidery, wood carving, etc. Special attention is paid to the needs of those learning to become illustrators. The practical articles also include "Drapery Upon the Human Figure," "Pen Studies of Heads," "Flower Drawing in Pen-and-Ink," "Flower Painting in Oil," "Flower Painting in Water Colors," "Portrait Painting," "Landscape Painting," and "Designing for Lithographers"—an excellent field.

